# Quality of life and associated factors among infertile women attending infertility clinic at Mnazi Mmoja Hospital, Zanzibar

**DOI:** 10.1186/s12905-023-02536-4

**Published:** 2023-08-01

**Authors:** Mubina Suleiman, Furaha August, Mary Winnie Nanyaro, Peter Wangwe, Amani Kikula, Belinda Balandya, Matilda Ngarina, Projestine Muganyizi

**Affiliations:** 1 Department of Obstetrics and Gynecology, School of Medicine, Muhimbili University of Health and Allied Sciences, P.O. Box 65001, Dar es salaam, Tanzania; 2 Department of Research, National Institute of Medical Research, P. O. Box 3436, Dar es salaam, Tanzania; 3 Department of Obstetrics and Gynecology, Muhimbili National Hospital, Malik Road, Upanga West, P. O Box 65000, Dar es Salaam, Tanzania; 4 Department of Obstetrics and Gynaecology, University of Dar es Salaam, Mbeya College of Health and Allied Sciences, Mbeya, Tanzania

**Keywords:** FertiQol, Infertile women, Quality of life, Factors

## Abstract

**Background:**

Worldwide, it is estimated at least 50 million couples are affected by infertility with the prevalence of infertility being 16% in Tanzania. Psychological impact of infertility in patients negatively affects women’s Quality of Life (QoL) defined as a person`s perception of where they are in life in terms of culture and value in the emotional, mind-body, relational, social, environment and tolerability of treatment aspects. Poor Quality of Life is related to increased treatment discontinuation. The aim of this study was to determine the Quality of Life and associated factors among infertile women attending infertility clinic at Mnazi Mmoja Hospital, Zanzibar.

**Methods:**

A hospital based cross–sectional study was conducted among 340 infertile women attending infertility clinic at Mnazi Mmoja Hospital, Zanzibar. Data was collected using FertiQoL tool. The factors associated with Quality of Life using FertiQoL tool in infertile women were estimated in a multivariable linear regression model at 95% confidence interval and 5% level of significance.

**Results:**

Quality of life of infertile women at Mnazi Mmoja infertility clinic was 70.6 ± 10.0 on a scale of 0 to 100. It increased significantly with increase in educational level (p = 0.009). Women with female individual causes on average had 5.07 (B=- 5.07, 95%CI: -7.78, -2.35) and women with individual and respective male partner causes of infertility had on average 4.95 (B= -4.95, 95% CI: -7.77, -2.12) respective decrease in the FertiQoL scores compared to those who had their male partner with problems as reason for infertility. There was an average 4.50 (B=-4.50, 95% CI: 2.30, 6.70) decrease in quality of life in women with secondary infertility compared to women with primary infertility. Every month increase in duration of infertility led to an average of 0.04 (B=-2.57, 95%CI: -0.07, -0.01) decrease in FertiQoL scores.

**Conclusion:**

The overall quality of life in this population was positively associated with level of education but negatively affected with reason for infertility, type of infertility and duration of infertility.

## Background

Worldwide, it is estimated that 45 million couples are affected by infertility and 186 million individual live with infertility [1–3]. The inability to bear children is a tragedy for many women and can cause uncomfortable emotional situations throughout life, psychological distress, discrimination, low self-esteem [4–7]. All these distressing and negative emotional experiences can lead to poor outcome of fertility treatment and it has been reported it can lead to stopping the pursuit of fertility treatment [8]. Infertile women also have lower Quality of Life compared to infertile men. The QOL is defined as a person`s perception of where they are in life in terms of culture and value in the emotional, mind-body, relational, social, environment and tolerability of treatment aspects [9, 10]. A systematic review on assessing quality of life utilizing different QoL tools including FertiQoL, Short Form Health survey 36 questionnaire (SF-36) and WHO-QOL among others showed that there is a decreased QoL scores on infertile women [11].

Social effects of childlessness are very severe for women in low-income countries such as Tanzania with a 16% prevalence of infertility [12]. A survey done in Northern Tanzania has shown the most detrimental consequence of infertility is lack of respect and stigma within the community that can be explained through high rates of depression among infertile women where having a child is a necessity. Not only does infertility affect health related domains that affect women`s QoL but also increases their treatment discontinuation [13, 14].

Infertility remains costly despite the call for Universal Health Coverage agenda by 2030 [15]. Moreover direct psychiatric effects manifesting as irritability, anxiety, depression, and psychosis [16] contradict with target 3.4 in the third Sustainable Development Goal that voices out promotion of mental health and well-being by 2030 [17]. Infertility care remains sparse and very expensive and can be stressful in Tanzanian health care centers. This study aimed to determine the QoL and associated factors in infertile women attending Mnazi Mmoja hospital in Zanzibar.

## Methods

### Study design and sampling

**Fig. 1:**
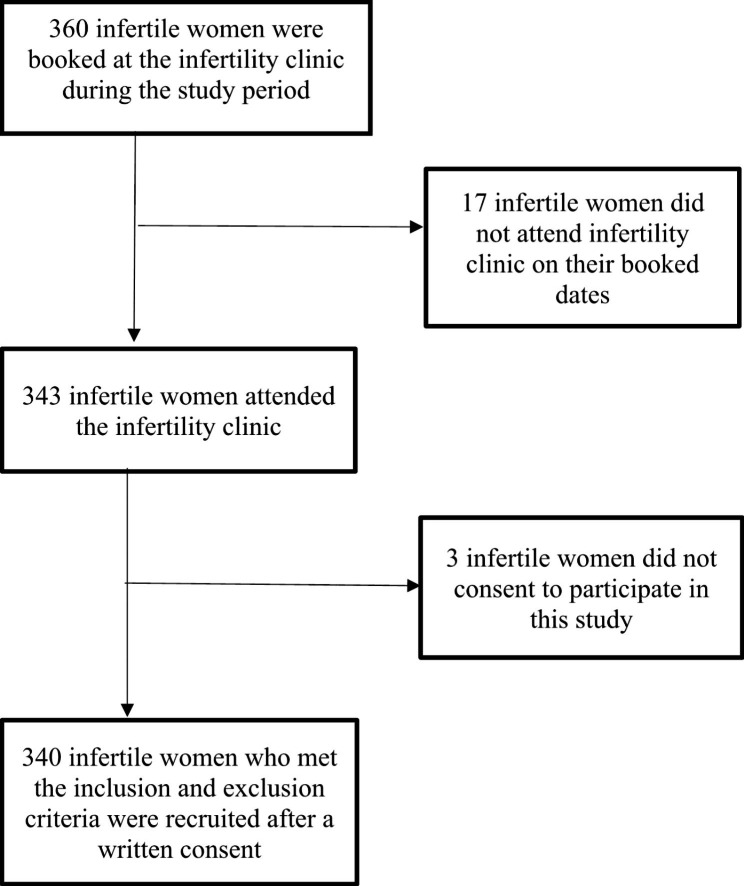
Recruitment flow chart of infertile women who participated in the study

This was a hospital based cross-sectional analytical study done from August 2020 to January 2021. The study was conducted at Mnazi Mmoja Hospital (MMH), Zanzibar. Mnazi Mmoja Hospital is a public referral hospital located in Stone town area, urban Unguja, Zanzibar. It is a teaching hospital for College of Health Sciences. It was chosen because it receives women referred from all district hospitals (Urban District, West, Central, South, North A and North B) in Unguja and Pemba. It is the only public hospital with an infertility clinic in Zanzibar. The study participants were chosen by non-probability method using convenience sampling, whereby all infertile women who met the inclusion criteria were enrolled until the estimated sample size was reached. Participants were recruited on their clinical appointment days. Infertility clinics are conducted at gynecologic clinic. The clinic was started in 2017, it runs every Tuesdays where approximately 15–30 infertile women are seen with 10–20 new and 5–10 follow up cases. Every participant had a unique number for identification and marked to avoid repetition in subsequent days of data collection. The sample size was calculated using the standard deviation from a study done in Uganda [18]. It was calculated using formula Z_1−α/2_^2^SD^2^/d^2^ [19] whereby Z_1−α/2_ was 2.33 and the margin of error was 2%. This was then multiplied by 1.2 design effect and later with infertility being a sensitive topic 30% non-response rate [20] was added that resulted to approximately 360 women. However, the final sample size was 340 infertile women well above the minimum sample size (Fig. 1).

### Data collection instrument

The FertiQoL scale is a reliable, validated, disease-specific international scale that measures QoL in both men and women experiencing infertility with core and treatment domains [10]. It yields six subscales and total scores with a range of 0 to 100. The Total FertiQoL score was the average QoL for all core and treatment domains. Cronbach reliability statistics used for this study in the Core and Treatment FertiQoL (and subscales) is satisfactory, in the range of 0.64 and 0.92 as seen in Table 1.

**Table 1:** FertiQoL scores according to different domains and their respective Cronbach reliability coefficients

Scale	Cronbach reliability coefficient	Mean ± SD
**Sub-Scales**		
Emotional Mind-Body Relational Social Treatment Tolerability Treatment Environment	0.62 0.56 0.56 0.53 0.68 0.66	77.4 ± 14.0 59.5 ± 19.8 50.8 ± 17.9 68.9 ± 15.5 72.1 ± 16.5 96.4 ± 10.7
FertiQoL Core	0.72	64.2 ± 12.6
FertiQoL Treatment	0.10	81.9 ± 11.1
Total	0.65	70.6 ± 10.0

All infertile women attending infertility gynecology clinic on Tuesday willing to participate were interviewed after obtaining informed consent, while those declared mentally unstable clinically were excluded.

Participants were identified through the infertility clinic registry book. The eligible participants were recruited after completion of clinical care services and provided with unique identification study numbers. The interview was carried out in two private rooms within the clinical building and confidentiality was maintained. Data was collected using a pre-tested Swahili version of FertiQoL questionnaire.

The principal investigator (PI) was assisted by two trained research assistants (RA), who were trained in a workshop that took a period of two days right before the piloting then commencement of the study. The PI trained the research assistants with practical sessions to ensure consistent understanding of the tool. The PI supervised the research assistants in the piloting and for a period of one month, then once in two weeks for the remaining four months’ data collection period. Their role was to interview participants together with the PI. The participants were allowed to ask questions during the interview for further clarifications that were answered by the research assistants and PI.

The PI went through the completely filled questionnaires daily and consulted with the RA for any errors ready for double entry. Pre-testing of the questionnaire was done at Mnazi Mmoja hospital, before commencing the research, in order to evaluate whether the questionnaire captured the intended objectives of the study and to estimate the time needed for a single interview in order to plan for data to be collected accordingly.

### Data Management

#### Variables

The FertiQoL score was treated as a continuous variable ranging from 0 to 100, with higher scores indicating a better QoL of the participant. The exposure variables were divided into social demographic and fertility related factors. The socio demographic factors included age in years which was categorized to ≤ 30 and ≥ 31 (34); marital status was categorized into Not in union (single, divorced, widow) and In union (cohabiting, married); education level was categorized into no formal education, primary, secondary and higher education; occupation was categorized into employed and unemployed, districts were categorized into urban, north, south, Pemba and others; religion was categorized into Muslim and Christian. The fertility related factors included were duration of infertility measured in months taken as a continuous variable; reason for infertility that was categorized into male, female, both male and female and idiopathic, type of infertility which was categorized into primary and secondary while any previous treatment received was categorized to no and yes.

### Data Analysis

Data was checked for completeness and correctness. Data was analyzed using STATA version 15 statistical software: (StataCorp. 2017. *Stata Statistical Software: Release 15*. College Station, TX: Stata Corp LLC). In descriptive statistics, QoL score was summarised using mean and standard deviations while duration of infertility was summarized using median and interquartile range. Categorical variable was summarized using frequency and proportion. Linear regression model was used to determine factors associated with QoL. Test for normality, homoscedasticity and linearity were conducted to test assumption of the linear regression model.

Bivariable analysis was used to determine the associations between the QoL and individual social demographic and clinical factors. Multivariable linear regression model was used to adjust for possible confounders. All variables with p values less than 0.2 were included in the multivariable model. All tests were two tailed and p-values of 0.05 were considered significant in the final model. Assessment of a parsimonious model was based on the lowest Akaike information criteria.

## Results

### Socio-demographic and clinical characteristics

A total of 340 infertile women were recruited from the infertility clinic at Mnazi Mmoja Hospital. Majority of the women, 325 (95.6%) were married, while 242 (71.2%) of women had secondary education, 190 (55.9%) had no employment. Among the 340 infertile women, 160 (47.1%) women were the reason of infertility among the couples. There were 202 (59.4%) women with primary infertility and the median duration for infertility was 48 (36–96) months. More than half of the women 234 (68.8%) had previous treatment (Table 2).

**Table 2:** General and clinical characteristics of the participants (N = 340)

Characteristics	n (%)
**Age (years)**	
≤30 ≥31 *Mean ± SD*	233 (68.5) 107(31.5) 29.3 ± 5.8
**Marital Status**	
Not in union In union	15 (4.4) 325 (95.6)
**Education**	
No formal education Primary education Secondary education Higher education	9 (2.6) 44 (13.0) 242 (71.2) 45 (13.2)
**District**	
Urban North South Pemba Others	252 (74.1) 30 (8.8) 52 (15.3) 3 (0.9) 3 (0.9)
**Religion**	
Muslim Christian	331 (97.4) 9 (2.6)
**Reason for infertility **	
Male Female Male & Female Idiopathic	75 (22.1) 160 (47.1) 77 (22.6) 28 (8.2)
**Type of infertility**	
Primary Secondary	202 (59.4) 138 (40.6)
**Duration of infertility (months)**	
*Median (IQR)*	48 (36–96)
**Any previous treatment received **	
No Yes	106 (31.2) 234 (68.8)

### Quality of life of infertile women using FertiQol tool

The overall mean fertility QoL score of the infertile women was 70.6 ± 10.0 with a total Cronbach reliability of 0.65. According to the subscales, the women on average had 77.4 ± 14.0 score for the QoL in the emotional domain, 59.5 ± 19.8 for the Mind-Body domain, 50.8 ± 17.9 for the relational domain and 68.9 ± 15.5 for the social domain. The mean core score on the FertiQoL was 64.2 ± 12.6. Moreover, the mean treatment tolerability score was 72.1 ± 16.5 and mean treatment environment score was 96.4 ± 10.7 which constituted the FertiQoL mean Treatment score of 81.9 ± 11.1 (Table 1).

### Factors Associated with Quality of Life of Infertile Women

In bivariable linear regression analysis education level and type of infertility were significantly associated with an average increase in QoL score compared to reason of infertility, duration of infertility and previous treatment which were significantly associated with an average decrease in QoL scores. In adjusted linear regression model as shown in Table 3 below the education level remained to be significantly associated with FertiQoL scores in the infertile women. Findings show that women with secondary and higher level of education had an average positive change in mean FertiQoL scores by 12.6 (B = 12.56, 95% CI 0.91, 24.22) and 16.7 (B = 16.71, 95% CI: 4.72, 28.70) respectively compared to those with no education. Trend test shows that mean QoL were significantly increased with the increase in level of education (P trend = 0.021).

**Table 3:** Socio demographic factors associated with quality of life of study participants at Mnazi Mmoja Hospital (N = 340)

Characteristics	Coefficient(95%CI)	p-value	Adjusted coefficients (95%CI)	p-value
**Age (in years)**				
≤ 30	1			
≥ 31	-1.43 (-3.78, 0.92)	0.233		
**Marital status**				
Not in union	1			
In union	-1.34 (-8.71, 6.02)	0.720		
**Education level**				
No education	1		1	
Primary level	9.56 (-2.60, 21.72)	0.068	11.00 (-0.98, 22.99)	0.072
Secondary level	10.50 (-1.30, 22.29)	0.081	12.56 (0.91, 24.22)	0.035
Higher level	13.52 (1.47, 25.58)	0.028	16.71 (4.72, 28.70)	0.006
**Occupation**				
Unemployed	1			
Employed	-0.41(-2.60, 1.79)	0.716		
**District**				
Urban	1			
North	-3.13 (-6.96, 0 0.71)	0.110		
South	-2.49 (-5.33, 0.34)	0.085		
Pemba	-12.01 (-35.84,11.82)	0.322		
Others	0.73 (-16.80, 18.25)	0.935		
**Religion **				
Muslim	1			
Christian	-11.63 (-23.34, 0.07)	0.052		
**Reason for infertility**				
Male	1			
Female	-4.29 (-6.95, -1.62)	0.002	-4.87 (-7.58, -2.17)	< 0.001
Male and Female	-4.14 (-6.98, -1.31)	0.004	-4.81 (-7.61, -2.01)	0.001
Idiopathic	-2.19 (-6.33, 1.95)	0.299	-1.73 (-6.42, 2.96)	0.468
**Type of infertility**				
Primary	1		1	
Secondary	2.97 (0.86, 5.07)	0.006	4.45 (2.30,6.60)	< 0.001
**Duration of infertility(months)**	-0.03 (-0.06, − 0.01)	0.016	-0.04 (-0.06, -0.01)	0.002
**Previous treatment**				
No	1		1	
Yes	-2.31 (-4.46, -1.17)	0.034	-2.34 (-4.45, -0 0.23)	0.030

After keeping all other factors constant, those who had female reasons for infertility and those who had both male and female reasons for infertility, on average, had 4.9 (B= -4.87, 95% CI: -7.58, -2.17) and 4.8 (B= -4.81, 95% CI: -7.61, -2.01) respective significant decreases in the FertiQoL scores compared to those who had male reasons for infertility among the couples. Women with secondary infertility had an average 4.5 (B = 4.45, 95% CI: 2.30, 6.60) significant increase in FertiQoL scores compared to women with primary infertility. Similarly, for every monthly increase in the duration of infertility there was an average 2.3 (B= -2.34, 95% CI: -4.45, -0 0.23)) decrease in the FertiQoL scores in the infertile women (Table 3).

## Discussion

The study found the mean total quality of life score to be 70.6 ± 10.0. The women had a lower FertiQoL core score of 64.2 ± 12.6 which included emotional, mind-body, relational and social domain compared to the treatment score of 81.9 ± 11.1. Factors associated with QoL of infertile women were higher level of education, reason of infertility, type of infertility and duration of infertility.

The Total quality of life findings are consistent with those in the cross sectional study done in 29 Dutch infertile clinics whose average FertiQoL score was 70.8 ± 13.9 and in Turkey on 174 infertile couples which showed the mean infertile women quality of life to be 70.0 ± 13.5 [21, 22]. This could be because a majority of the women were educated at secondary and higher level of education hence knowledgeable on the presence of infertility. However, these results are relatively higher compared to those seen in infertile and fertile women in Iran and Uganda [23, 24].

One of the socio-demographic factors that was significant and positively associated with QoL of infertile women included higher level of education which is similar to other studies [22, 25]. This could be because people of higher education are less embarrassed than those with lower education. Moreover, people with higher education level use better problem-solving skills, learn how to deal with daily stressors and use creative solutions to deal with new problems.

Age was not significantly associated with FertiQoL scores in this study. Similarly, other studies found no significant association between age and QoL [26, 27] contrary to a study done in Turkey, where younger women were associated with low scores of emotional, social and core domains [22]. However, in this study majority of the women were equal to or less than 30 years of age and therefore might be still hopeful of getting children, while those who were 30 years and above have already found coping mechanisms with infertility as time passes by.

Secondary type of infertility was associated with higher FertiQoL scores as seen in other studies where certain domains of the FertiQoL had a more positive association with secondary infertility compared to primary infertility [28, 29]. This was contrary to some studies which reported no significant relationship between the QoL and the type of infertility either being primary or secondary [26, 28]. As secondary infertility occurs later in life, women with secondary infertility have less time suffering from psychological stress, stigma and other depressors compared to those with primary infertility.

This study showed that QoL of infertile women decreased significantly when the reason for infertility was, the woman alone or both the man and the woman. These results could be due to social cultural norms surrounding women in Zanzibar where if the couple does not have children the society sees the woman as a failure. Moreover, the men are allowed to marry more than one wife so the women feel insecure and hence susceptible to a worse QoL compared to men.

QoL of the infertile women was shown to decrease with an increase in the duration of infertility. This could be because of the infertile women losing hope after trying different solutions that didn`t work and being surrounded by societal pressure throughout their duration of infertility. This is similar to studies that pointed out prolonged duration of infertility had an adverse impact on the QoL of women [28, 30] although some studies showed no significant association between duration of infertility on QoL of infertile women [25, 31]. However, this is against a significant negative association seen between the total and core (emotional, mind-body, and social subscales) scores of the FertiQoL and the attempted duration of trying to conceive [30].

The study limitations included a threatened external validity of this hospital-based study since not all women who are infertile come to the infertility clinic hence, we cannot generalize these results to the general population. In addition to that, the cross-sectional study design limited our ability to make causal inferences on the QoL of infertile women and infertility. The infertile women at Mnazi Mmoja infertility clinic should regularly be asked the FertiQoL tool questions so as to deduce their overall QoL scores and scores in specific domains as we now have the overall score and scores of the domains for further research. The focus of counselling activities at the infertility clinic in Mnazi Mmoja Hospital, should consider factors such as education, reason of infertility, type of infertility and duration of infertility when managing these infertile women.

## Conclusion

The QoL of infertile women at the infertility clinic in Mnazi Mmoja Hospital according to the FertiQoL scores is satisfactory. The socio demographic factor significantly associated with the QoL is level of education while the clinical factors significantly associated with QoL include reason of infertility, type of infertility and duration of infertility.

## Data Availability

The datasets used and/or analysed during the current study are available from the corresponding author on reasonable request.

## List of Abbreviations

FertiQoL: Fertility Quality of Life
MUHAS: Muhimbili University of Health and Allied Sciences
QoL: Quality of Life
WHO: World Health Organization
